# Evaluation of anthelmintic potential of the Ethiopian medicinal plant *Embelia schimperi* Vatke *in vivo* and *in vitro* against some intestinal parasites

**DOI:** 10.1186/s12906-015-0711-7

**Published:** 2015-06-18

**Authors:** Yared Debebe, Mesfin Tefera, Walelign Mekonnen, Dawit Abebe, Samuel Woldekidan, Abiy Abebe, Yehualashet Belete, Temesgen Menberu, Bethelhem Belayneh, Berhanu Tesfaye, Ibrahim Nasir, Kidist Yirsaw, Hirut Basha, Asrat Dawit, Asfaw Debella

**Affiliations:** Ethiopian Public Health Institute, Biomedical and Clinical Research Team, Traditional and Modern Medicine Research Directorate, P.O.Box 1242, Addis Ababa, Ethiopia; Ethiopian Public Health Institute, National Polio Laboratory, P.O.Box 1242, Addis Ababa, Ethiopia; Ethiopian Public Health Institute, Natural products Research Team, Traditional and Modern Medicine Research Directorate, P.O.Box 1242, Addis Ababa, Ethiopia; College of Health Sciences, Addis Ababa University, P.O.Box 9086, Addis Ababa, Ethiopia

**Keywords:** Anthelmintics, Cestocidal, Embelin, *Hymenolepis nana*

## Abstract

**Background:**

*Embelia schimperi* has been used for the treatment of intestinal parasites especially tapeworm infestations for centuries in Ethiopia. However, there is lack of scientific based evidences regarding the efficacy, safety and phytochemical analysis of this plant despite its frequent use as an anthelmintic. This study has therefore evaluated the efficacy and acute toxicity of *E. schimperi* thereby generating relevant preclinical information.

**Methods:**

The anthelmintic activities of the crude hydroalcoholic extract of *E. schimperi* and the isolated compound, embelin, were conducted using *in vivo* and *in vitro* models against the dwarf tapeworm, *Hymenolepis nana*, and the hookworm, *Necator americanus*, respectively. LD_50_ of the crude hydroalcoholic extract was determined using Swiss albino mice following the OECD guidelines. Chemical characterization of the isolated embelin was conducted using UV-spectroscopy, HPLC and NMR.

**Results:**

In the acute toxicity study no prominent signs of toxicity and mortality were recorded among the experimental animals at the highest administered dose. Hence the LD_50_ of the plant was found to be higher than 5000 mg/kg. *In vivo* cestocidal activity of the crude hydroalcoholic extract of *E. schimperi* showed 100 % parasite clearance at 1000 mg/kg, while the diammonium salt of embelin showed 85.3 % parasite clearance at 750 mg/kg. The *in vitro* anthelminthic activity study revealed that the LC_50_ value of the crude extract and albendazole were 228.7 and 51.33 μg/mL, respectively.

**Conclusion:**

The results clearly indicated that the hydroalcoholic extract of *E. schimperi* and the diammonium salt of the isolated compound embelin had anthelmintic activity against hookworm larva *in vitro* and *H. nana in vivo*. Hence the findings of this study showed *Embelia schimperi* appears to possess some anthelmintic activity that may support the usage of these plants by local traditional healers to treat helminthic infestations.

## Background

Helminthiases have been affecting human beings throughout the history of mankind and still continue to be major cause of mortality and morbidity to over a billion people in the world, particularly in developing regions like the sub-Saharan Africa (SSA), Asia and the Americas. These regions of the world are characterized by marginalized societies with resource constraints making the burden more vicious exacerbating other health and socioeconomic problems like malaria, HIV/AIDS and decreased productivity [1]. The Ethiopian scenario is not different from other SSA countries as the Federal Ministry of Health of Ethiopia (FMoH) [2] reported that annual visits of more than half a million cases in the outpatient departments of health facilities are as a result of intestinal parasitic infections including helminthiases. This number might not represent the actual burden as some of the health facilities lack the appropriate diagnostic methods as well as failure of detecting lower parasite burden [3].

The Ethiopian custom of using medicinal plants for the treatment of intestinal parasites has existed for many generations. Quite many numbers of herbal remedies are prescribed in the traditional health care system in this regard. *Embelia schimperi*, a plant which belongs to family Myrsinaceae, is among the most widely used anthelmintic medicinal plant in Ethiopian folk medicine. Various ethnomedical studies revealed that the fruit is widely consumed to expel the adult stage of beef tapeworm and other intestinal parasites from the body [4–7]. Despite this frequent utilization of the plant as an alternative anthelmintic medicine, scientific evidences are less abundant supporting the traditional claims in Ethiopia. This study was therefore evaluated the acute toxicity and anthelmintic properties of the most frequently used medicinal plant*, Embelia schimperi* Vatke.

## Methods

### Plant collection and authentication

Fruits of *Embelia schimperi* were collected from the localities in Gonder, Northwest Ethiopia. A voucher specimen (ES-2175) was collected and deposited at the herbarium of Traditional and Modern Medicine Research Directorate (TMMRD), Ethiopian Public Health Institute (EPHI) following its authentication by the taxonomist of the research directorate.

### Experimental animals

The animals used in this study were the Swiss albino mice, *Mus musculus albinus.* The animals were obtained from the animal breeding unit of EPHI and kept in standard cages in the animal house of TMMRD. They were fed with standard pellet diet and tap water *ad libitum* and maintained at temperature of 21 ± 2 °C and humidity of 65 ± 0.5 % with 12 h light/dark cycles until the end of the experiment. All experimental animals were acclimatized for 10 days prior to the experimental procedures.

### Preparation of crude extract

The air dried fruits of *E. schimperi* were pulverized into powder form using an electronic grinding mill. The fruit powder (200 g) was extracted in 80 % ethanol by maceration. The extract was then filtered with Whatman filter paper No 3 and the organic solvent was removed from the filtrate using rotary evaporator (BUCHI B-205, Switzerland). The aqueous residue was further lyophilized (Labconco, USA) resulting in 15.79 g of crude extract.

### Isolation of embelin from *E. schimperi* and preparation of diammonium salt of embelin

Isolation of the reference compound, embelin, was conducted following the procedure described by Belete et al. [8]. Fruit powder of *E. schimperi* weighing 1.1 Kg was extracted using ethyl-acetate for 25 min and then transferred to a shaker for 4 h and filtered with Whatman filter paper No. 41. The marc was extracted one more time with the same solvent and followed by evaporation under vacuum yielding 119.71 g. The resulting extract was successively washed using hexane which resulted 5 g of an orange crystal of pure embelin having a melting point of 142–143 °C whose identity was fully elucidated by HPLC, ^1^H, ^13^C, DEPT-135, HMQC, HMBC and UV spectra. The pure isolated embelin was then dissolved with methanol and ammonium was added until the solution turned into pink followed by evaporation to produce diammonium salt of embelin. The salt was completely soluble in water and easy for administration to the experimental animals as opposed to the pure embelin which was totally insoluble in water.

### Acute toxicity study and determination of LD_50_

The acute toxicity of the crude extract was determined following Lorke’s [9] method. The crude fruit extract was administered orally at increasing doses of 625, 1250, 2500, and 5000 mg/kg, p.o. to six animals in each group. The general signs and symptoms of toxicity such as death, changes in physical appearance or behavioral changes were observed for 24 h post administration of the extract. The median lethal dose (LD_50_) was calculated as geometric mean of the dose that resulted in 100 % lethality and the maximum dose with no lethality at all.

### *In vitro* parasite culturing of hookworm eggs

The filter paper test tube culture technique following Harada and Mori [10] was used for the development of larvae from egg infested stool. Stool samples were collected in Wolkite health center after verbal consent was obtained from the patients. About 1 g of fecal matter was applied on a filter paper which was then inserted in a test tube containing water. The samples were then incubated under room temperature for approximately ten days. Monitoring of larval development of *N. americanus* was conducted until the desired larval stage was achieved for the efficacy study. After obtaining the infective larval stage the samples were centrifuged at a speed of 402 × g for ten minutes and concentrated. The number of larvae present was adjusted to achieve a concentration of one larva per microliter.

### *In vitro* anthelmintic activity

The 96-well microtiter plate assay described by Gill and others [11] was followed to evaluate the effects of the crude extract on the third stage larvae of *N. americanus*. Stock solutions of the crude extract of *E. schimperi* and albendazole (Sigma-Aldrich), the standard drug serving as a positive control, were prepared at 100 and 20 mg/mL, respectively, in 1 % dimethylsulfoxide (DMSO) and were serially diluted by two-fold to produce a series of dilutions. Aliquots were added at a dilution of 1 % to molten nutrient agar in a total volume of 200 μL in individual wells of a 96-well microtiter plate. The final concentrations in the assay plates consisted of two-fold serial dilutions starting 1, 0.5, 0.25, 0.125 and 0.0625 mg/mL for the plant extract and 0.2, 0.1, 0.05, 0.025 and 0.0125 mg/mL for the standard drug, albendazole. Approximately 30 larvae in 30 μL of distilled water were added to each well, and the plate was incubated in the dark at 25 °C for 48 h.

The effect of the drugs on worm viability was assessed by counting the numbers of motile larvae after the 48 h of incubation period. The larvae were stimulated to move by addition of 40 μL of water warmed at 50 °C to each well, control larvae and those unaffected by the extract and the standard drug were observed to move in a rapid sinusoidal motion. In contrast, extract and drug affected larvae showed a twitching motion or remained motionless. Individual larva moving with sinusoidal motion was counted. All assays were performed using triplicate assay wells at each drug concentrations.

### *In vivo* anticestodal activity

The anticestodal activities of the test extracts were conducted on experimentally infected Swiss albino mice which were maintained in the laboratory. Adult worms of *H. nana* were collected from the intestines of infected mice. Eggs were collected from the worm through dissection and the dose of infection was adjusted to be 800 eggs in 0.1 ml of normal saline. Each experimental mouse was then infected orally by stomach tube. Two weeks post infection, fresh fecal samples from each infected mice were collected and examined for shedding of ova. Mice not shedding ova of *H. nana* were discarded from the experiments.

The mice were then divided randomly into five groups of six mice each for each test extract. The three groups of animals were assigned as the test groups whereas the other two groups were used as control (positive and negative). Animals in the positive control group were treated with the standard drug Praziquantel at 25 mg/kg body weight whereas the negative control received equal volume of the vehicle. The other three groups were used for evaluation of the Anticestodal activities of the test extracts. 48 h post extract and drug administration; all the mice were sacrificed to determine percent deparasitization [12].

$$ \%\ \mathrm{deparasitization}=\left(\mathrm{N}-\mathrm{n}\right)/\mathrm{N}\times 100 $$

*N* = numbers of worms counted in the negative control group

*n* = number of worms counted in the plant extract or Praziquantel treated mice

Percent host clearance was determined using the following formula

$$ \%\ \mathrm{host}\ \mathrm{clearance} = \mathrm{A}/\mathrm{B}\times 100 $$

A = number of hosts found devoid of worms

B = number of hosts originally infected with worms

### Statistical analysis

Determination of LC_50_ of a sigmoidal concentration response curve was performed using GraphPad Prism version 6.04 for Windows (GraphPad Prism®; GraphPad Software, Inc., San Diego, California, USA). Statistical significance for *in vivo* anthelmintic activity effect of the extracts was determined using one way ANOVA and LSD multiple comparison tests. *P* value <0.05 was considered significant.

### Ethical considerations

Ethical approval for collection of patient stool samples and use of experimental animals for the experimental procedures was obtained from the Scientific and Ethical Review Office, EPHI (006/2003). Patients’ consent was sought in verbal form. Those people with confirmed hookworm infection were treated with albendazole. Animals were handled with care as per the guideline of EPHI for animal handling.

## Result

### Isolation of embelin from *E. schimperi*

**Fig. 1:**
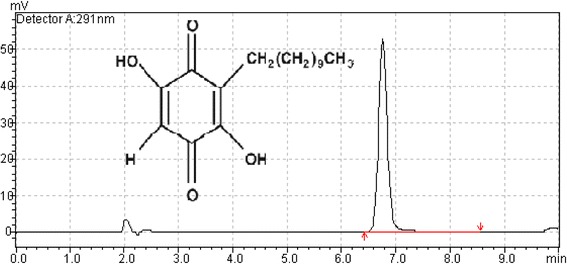
High performance liquid chromatogram of the isolated embelin

The compound isolated was embelin confirmed by NMR spectroscopy. ^1^H-NMR and ^13^C-NMR spectra of the purified sample verified the identity of the compound to be embelin. ^1^H-NMR (400 MHz, CDCl_3_) δ: 7.68 (s, 2H, OH), 6.00 (s, 1H, H-6), 2.44 (t, 2H, H-1^1^, *J* = 6.9 Hz), 1.47 (m, 2H, H-2^1^) 1.25-1.30 (m, 16H, H-3^1^ to 10^1^), 0.88 (t, 3H, H-11^1^, *J* = 6.9 Hz); ^13^C-NMR (100 MHz, CDCl_3_) (3, 116.99), (6, 102.15), (1^1^-10^1^, 22.51–31.90), (11^1^, 14.09). The HPLC profile of the purified embelin is shown in Fig. 1.

### Acute toxicity study and LD_50_ determination

No prominent sign of toxicity and mortality was recorded among experimental animals at all administered dose levels of the crude hydroalcoholic extract of *E. schimperi*. Since no death was recorded at the maximum administered dose, the LD_50_ of the plant was found to be higher than 5000 mg/kg.

### *In vitro* larval mortality assay

The crude hydroalcoholic extract of *E. schimperi* showed larvicidal activity against the infective stage (L_3_) of hookworms. The best-fit LC_50_ values for the crude extract and the standard drug, albendazole are indicated in Table 1. The LC_50_ value of the crude extract and albendazole were 228.7 and 51.33 μg/mL, respectively.

**Table 1:** LC_50_ of the crude extract and albendazole using global sigmoidal model curve fitting

	Log LC_50_		LC_50_ (μg/mL)		R^2^
	Best-fit	Std. error	Best-fit	95 % CI	
Crude extract of *E. schimperi*	2.359	0.0328	228.7	194.2 to 269	0.9402
Albendazole	1.710	0.0343	51.33	42.78 to 61.59	0.9583

### *In vivo* anticestodal activity

The result of *in vivo* anticestodal activity of the crude hydroalcoholic extract of *E. schimperi* and the diammonium salt of embelin is presented in Tables 2 and 3, respectively. The crude extract significantly (p <0.05) reduced the worm count at necropsy as compared with the negative control. The percent deparasitization and percent parasite clearance of mice treated with 1000 mg/kg were both 100 %. The mice in the negative control group had mean parasite count of 2.00 ± 0.32 with no single mouse devoid of worms at necropsy.

**Table 2:** Worm count, percent deparasitization and host clearance after treatment with crude extract of *E. schimperi*

Groups	Dose administered (mg/kg)	Mean no of parasites	% deparasitization	% host clearance
Group I	750	1.2 ± 0.49^*a^	40	40
Group II	1000	0.0 ± 0.0^*b^	100	100
Group III	2000	0.0 ± 0.0^*b^	100	100
Group IV	25 (PQ)	0.0 ± 0.0^*b^	100	100
Group V	vehicle	2.00 ± 0.32	0	0

*PQ*: Praziquantel

* Mean number of parasite within row is significantly different at *p* <0.05, while the letters ^a b^ show significant difference at *p* <0.01 as determined by LSD multiple comparison test

**Table 3:** Worm count, percent deparasitization and host clearance after treatment with diammonium salt of Embelin

Groups	Dose administered (mg/kg)	Mean no of parasites	% deparasitization	% host clearance
Group I	250	1.43 ± 0.30^*^	70.6	0
Group II	500	1.00 ± 0.69^*^	79.4	57.14
Group III	750	0.71 ± 0.36^*^	85.3	57.14
Group IV	25 (PQ)	0.00 ± 00^*^	100	100
Group V	vehicle	4.86 ± 1.18	0	0

*PQ*: Praziquantel

* Mean number of parasites within row is significantly different at *p* <0.05 determined by LSD multiple comparison test

Mice treated with 250, 500 and 750 mg/kg of diammonium salt of embelin had average worm count of 1.43 ± 0.30, 1.00 ± 0.69, 0.71 ± 0.36, respectively, at necropsy. While the mean worm count for the negative control group was 4.86 ± 1.18. The highest percent deparasitization and host parasite clearance, 85.3 % and 57.14 %, respectively was recorded in mice treated with the highest dose, 750 mg/kg.

## Discussion

In the present study single administration of the crude extract of *E. schimperi* up to a dose of 5,000 mg/kg to mice showed no behavioral changes like hypoactivity, prostration and pileroerection. The following day after the treatment, all mice were as active as the controls and no death was observed in the treated animals. The LD_50_, being greater than 5000 mg/kg, is thought to be safe as suggested by Lork (1983) and Schorderet (1992) [9, 13]. Again, the absence of death among mice in all the dose groups throughout the two weeks of the experimental follow up seems to support this claim. Thus, the crude extract of *E. schimperi* can be considered as a substance with low toxicity.

The crude hydroalcoholic extract of the fruits of *E. schimperi* and the diammonium salt of the isolated embelin exhibited significant anthelmintic activities against the dwarf tapeworm, *H. nana* and hookworm, *N. americanus*, *in vivo* and *in vitro*, respectively. Our finding is in agreement with various authors who reported that many plant species in family Myrsinaceae showed substantial anthelmintic properties. For instance, Choudhary [14] reported that the ethanolic extract of the seeds of *Embelia ribes* showed significant anthelmintic efficacy against the roundworm *Rhabditis pseudoelongata* (strain L. Lamy) *in vitro* when compared to the standard drugs, levamisole and ivermectin. Furthermore, Jalalpure *et al.* [15] evaluated the efficacy of the seed oil of *E. ribes* against *Pheritima posthuma* to determine the time of paralysis and time of death and found out that the seed oil was found to be more potent in both parameters than the standard drug reference, piperazine citrate. Two plant species in family Myrsinaceae, *Myrsine africana* and *Maesa lanceolata*, have been evaluated for their efficacy against *Haemonchus contortus* in Ethiopia. *In vitro* ovicidal and larvicidal activity studies of the two plants revealed that significant egg hatching inhibition and larvicidal effects were exhibited by different extracts of the plants [16, 17].

Embelin was isolated from the fruits of *E. schimperi* and its identity was confirmed by recording IR, MS and NMR spectra and by comparison of the spectral data with those reported by other authors [18, 19]. The diammonium salt of embelin showed significant anthelmintic activity *in vivo* against the flat worm *H. nana* with 85.3 and 57.14% deparasitization and host clearance, respectively. Similar finding have been reported regarding the anthelmintic properties of embelin. Gupta *et al.* [20] indicated that embelin had an anthelmintic activity *in vitro*. Mojumder and Mishra [21] reported the nematicidal activities of embelin and its two haloderivatives against root-knot nematode, *Meloidogyne incognita*. Embelin was efficacious at 48 and 72 h of exposure, and the haloderivatives were nematicidal even at 24 h post exposure. Bøgh *et al.* [22] evaluated the anthelmintic efficacy of diammonium salt of embelin *in vivo* and *in vitro* against the cestode parasites (*H. diminuta* and *H. microstoma*), the treamatode *Echinostoma caproni* and the nematode *Heligmosomoides polygyrus*. It significantly reduced the parasite number and total worm biomass of *H. diminuta* in rats treated with 100 mg/kg. *In vitro* studies also revealed that all adult *H. diminuta* were killed when incubated in culture medium with 0.08 mg/ml of diammonium salt of embelin. However, the *in vivo* studies on the other test parasites had no significant effect though the worms were inhibited *in vitro* suggesting that embelin might be more effective against cestodes than other intestinal parasites.

## Conclusion

In this study the possible anthelmintic efficacy of the traditionally claimed medicinal plant, *E. schimperi*, was investigated. The results clearly indicated that both the crude extract and the diammonium salt of the isolated embelin were effective anthelmintics against the dwarf tapeworm, *H. nana*, on mice at higher doses. Hence the findings of this study showed *E. schimperi* appear to possess some anthelmintic activity that may support the usage of this plant by local traditional healers to treat helminthic infestations. However, these higher doses might not be directly extrapolated to human use for health related issues and therefore further studies to optimize the dose for human use need to be conducted. Additional studies also need to be conducted on the mechanism of action, long term toxicity, and efficacy on other experimental models including human and standardization before it can be recommended for human use.

## Abbreviations

AIDS: Acquired immuno deficiency syndrome
ANOVA: Analysis of variance
DMSO: Dimethyl sulfoxide
EPHI: Ethiopian public health institute
FMoH: Federal ministry of health
HIV: Human immunodeficiency virus
HPLC: High performance liquid chromatography
LSD: Least significant difference
NMR: Nuclear magnetic resonance
OECD: Organization for economic co-operation and development
TLC: Thin layer chromatography
TMMRD: Traditional and modern medicine research directorate
UV: Ultraviolet
